# Obstructive Sleep Apnea and Cerebral Microbleeds in Middle-Aged and Older Adults

**DOI:** 10.1001/jamanetworkopen.2025.39874

**Published:** 2025-10-28

**Authors:** Ali Tanweer Siddiquee, Yoon Ho Hwang, Soriul Kim, Seung Ku Lee, Min-Hee Lee, Hyeon Jin Kim, Young Jin Kim, Bong-Jo Kim, Peter N. Hadar, M. Brandon Westover, Robert J. Thomas, Nan Hee Kim, Chol Shin

**Affiliations:** 1 Institute of Human Genomic Study, College of Medicine, Korea University Ansan Hospital, Ansan, Republic of Korea; 2 Institute of Human Genomic Study, College of Medicine, Korea University, Seoul, Republic of Korea; 3 Department of Paramedicine, Seowon University, Cheongju, Chungbuk, Republic of Korea; 4 Department of Neurology, Asan Medical Center, Seoul, Republic of Korea; 5 Division of Genome Research, Center for Genome Science, National Institute of Health, Chung cheong buk-do, 28159, Republic of Korea; 6 Department of Neurology, Massachusetts General Hospital, Boston, Massachusetts; 7 Department of Neurology, Beth Israel Deaconess Medical Center, Boston, Massachusetts; 8 Division of Sleep Medicine, Harvard Medical School, Boston, Massachusetts; 9 Division of Pulmonary Critical Care and Sleep Medicine, Department of Medicine, Beth Israel Deaconess Medical Center, Boston, Massachusetts; 10 Division of Endocrinology and Metabolism, Department of Internal Medicine, Korea University Ansan Hospital, Ansan, Republic of Korea

## Abstract

**Importance:**

The association of obstructive sleep apnea (OSA) with risk of incident cerebral microbleeds (CMBs) is unknown.

**Objective:**

To investigate the association between OSA severity and risk of incident CMBs in the late middle-aged general population.

**Design, Setting, and Participants:**

This cohort study included eligible participants who had in-home overnight polysomnography data and brain magnetic resonance imaging done at baseline (2011-2014) and 4-year follow-ups (first follow-up, 2015-2018, and second follow-up, 2019-2022) from an ongoing longitudinal cohort study of a Korean community of adults. Data were analyzed from March 2024 through January 2025.

**Exposures:**

OSA severity was categorized by apnea-hypopnea index levels as no OSA (0-4.9 events/h), mild OSA (5.0-14.9 events/h), and moderate to severe OSA (≥15.0 events/h).

**Main Outcomes and Measures:**

CMBs were defined as well-defined focal areas (<10 mm in diameter) of very low signal intensity on gradient echo T2*-weighted images. Modified Poisson regression (robust error variance) was used to estimate relative risk (RR), with 95% CIs, of incident CMBs by OSA group; adjustment was done for age, sex, education level, body mass index, physical activity level, smoking and drinking status, total and low-density lipoprotein cholesterol level, hypertension, diabetes, age-related white matter changes, change in apnea-hypopnea index level, change in body mass index, and mean arterial pressure of the corresponding follow-up.

**Results:**

Of 1441 study participants (mean [SD] age, 57.75 [5.53] years; 759 female [52.67%]), 436 participants (30.25%) and 193 participants (13.39%) had mild and moderate to severe OSA at baseline, respectively; 812 participants had no OSA. The cumulative incidence rate of CMBs in non-OSA, mild OSA, and moderate to severe OSA groups was 15 participants (1.85%), 7 participants (1.61%), and 9 participants (4.66%), respectively, at 4 years and 27 participants (3.33%), 14 participants (3.21%), and 14 participants (7.25%), respectively, at 8 years. In multivariable modified Poisson models, participants with moderate to severe OSA compared with the non-OSA group had an increased risk of developing CMBs at 8 years (RR, 2.14; 95% CI, 1.08-4.23; *P* = .02). These results were unaffected by the presence or absence of *APOE-ε4* carrier status. No significantly increased risks were observed at 4 years or in the mild OSA group at any time.

**Conclusions and Relevance:**

In this study, moderate to severe OSA was independently associated with an increased risk of incident CMBs over an 8-year follow-up. These results add to the evidence for the importance of sleep apnea to brain health.

## Key Points

**Question:**

Do middle-aged and older adults with obstructive sleep apnea (OSA) have increased risk of incident cerebral microbleeds (CMBs)?

**Findings:**

In this cohort study of 1441 adults, participants with moderate to severe OSA had an associated increased risk of developing CMBs compared with a non-OSA group over an 8-year follow-up.

**Meaning:**

This finding suggests that moderate to severe OSA may be an independent risk factor associated with incident CMBs in the general adult population.

## Teaser

This cohort study investigates the association of obstructive sleep apnea severity with risk of incident cerebral microbleeds in middle-aged and older adults in Korea.

## Introduction

Cerebral microbleeds (CMBs), hypointense ovoid lesions seen on gradient-recalled echo or susceptibility-weighted imaging due to blood-degradation products in the cerebral parenchyma, are known to constitute one of the early markers of cerebral vasculopathy.^1,2,3^ As a precursor, these CMBs are associated with increased risk of developing symptomatic stroke and dementia.^4,5^ The prevalence of CMBs in the general population varies from 3% in middle-aged individuals to as high as 23% in older individuals.^6^ It can increase to up to 50% to 70% in patients with cerebrovascular disease. Alarmingly, the incidence of stroke is increasing in relatively young and middle-aged people globally, which runs counter to the Sustainable Development Goal 3.4 to reduce the burden of stroke, part of the larger target to reduce the burden of noncommunicable diseases by one-third by 2030.^7^ In recent years, cognitive outcomes associated with CMBs have been highlighted as studies reported their associated risk with accelerated cognitive decline and dementia in the general population.^4,8^ However, to date, the only known modifiable risk factors associated with CMBs include smoking, hypertension, dyslipidemia, diabetes, and the presence of cardiocerebrovascular diseases.^9,10,11,12^

Obstructive sleep apnea (OSA) is a common chronic disorder characterized by repetitive collapse of the upper airway during sleep, associated nocturnal hypoxia, and sleep fragmentation.^13^ The disorder has been found to be highly correlated with cardiocerebrovascular diseases, such as arrhythmia, myocardial infarction, cerebral small vessel disease, and stroke.^14,15,16,17^ However, its particular association with CMBs is not yet well-established. Only a handful of studies have so far investigated OSA and its association with CMBs, and results have been mixed. For instance, a cohort study performed in 97 community-dwelling older adults^18^ showed that OSA was not associated with silent CMBs, and Song et al^19^ reported that moderate to severe OSA was not associated with CMBs based on an observational study in 175 patients at a sleep center referred for suspected OSA. On the other hand, moderate to severe OSA was found to be independently associated with CMBs in 75 patients undergoing polysomnography (PSG) in a hospital setup who were examined cross-sectionally.^20^ However, large meta-analyses^16,21,22^ investigating the association between OSA severity and CMBs have not found any associations, possibly because of scant existing data.

To our knowledge, no study to date has investigated the association between OSA and risk of incident CMBs longitudinally. Therefore, we aimed to examine associations between OSA severity and risk of CMBs in a large prospective cohort of late middle-aged through older individuals in the general population.

## Methods

Written informed consent was obtained from all participants in this cohort study, and the study protocol was approved by the institutional ethics committee of Korea University Ansan Hospital. We followed the Strengthening the Reporting of Observational Studies in Epidemiology (STROBE) reporting guideline.

### Study Design and Participants

**Figure 1.**
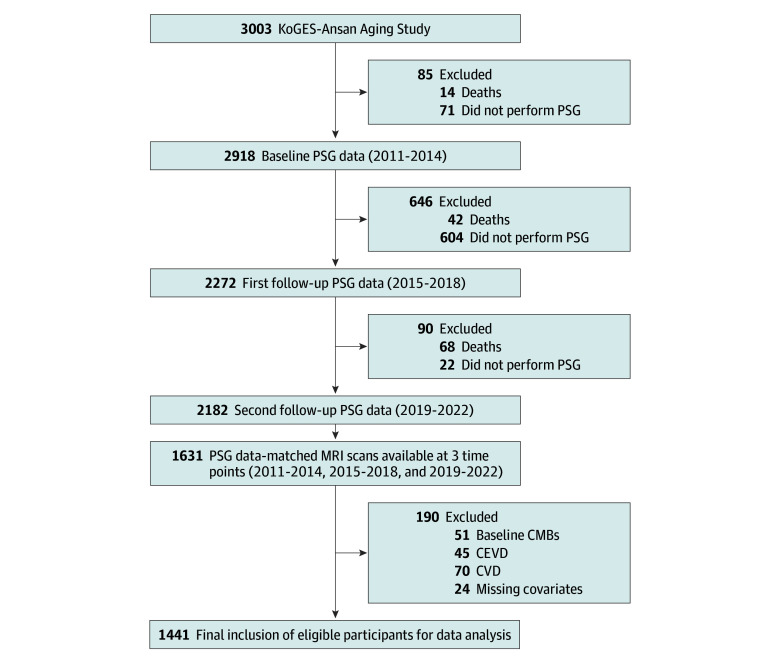
Study Flowchart CEVD indicates cerebrovascular disease; CVD, cardiovascular disease; KoGES, Korean Genome and Epidemiology Study; MRI, magnetic resonance imaging; PSG, polysomnography.

Study participants are from the Korean Genome and Epidemiology Study (KoGES), an ongoing prospective investigation of the population (Figure 1) that uses overnight, in-home polysomnography (PSG). The KoGES-Ansan Aging Study is a subcohort of KoGES. Details of the KoGES-Ansan Aging Study and sampling method have been provided in previous reports.^23,24^ Briefly, at the baseline examination between 2011 and 2014, a total of 2918 participants (mean [SD] age, 59.24 [6.89] years) underwent home-based, unattended PSG. The study population was then followed up at 4-year intervals with a scheduled site visit for similar interviews, comprehensive health examination, and collection of biospecimens. Two follow-ups were performed within years 2015 to 2018 and 2019 to 2022, when 2272 and 2182 participants, respectively, had PSG performed. Study participants also underwent structural brain magnetic resonance imaging (MRI) examinations at the same points. A total of 1631 participants were found to have PSG and MRI data at the 3 times (Figure 1). After exclusion of 51 participants who had baseline microbleeds, 45 participants with a history of cerebrovascular disease, 70 participants with a history of cardiovascular disease, and 24 participants with missing covariates, a total of 1441 participants were eligible for final analyses.

### Polysomnography

Overnight PSG was performed with a portable device (Embletta X-100; Embla Systems) at home. A trained technologist connected the device to the patient at bedtime, and data were retrieved in the morning after the unattended overnight recording. All PSG results were manually scored using standard criteria.^25^ Details of PSG are provided in our previous study and the eMethods in Supplement 1.^26^ Briefly, apnea was defined when airflow decreased by 90% of the baseline or greater for at least 10 seconds with ongoing respiratory efforts, and hypopnea was scored when at least 30% reduction of airflow for 10 or more seconds was accompanied by 4% or greater oxygen desaturation. Apnea-hypopnea index (AHI) level was calculated by finding the mean of the total number of obstructive apneas and hypopneas per hour of sleep. The change in AHI from baseline to follow-ups (change in AHI) was calculated (change in AHI = AHI at follow-up − AHI at baseline).

### MRI Data Acquisition

**Figure 2.**
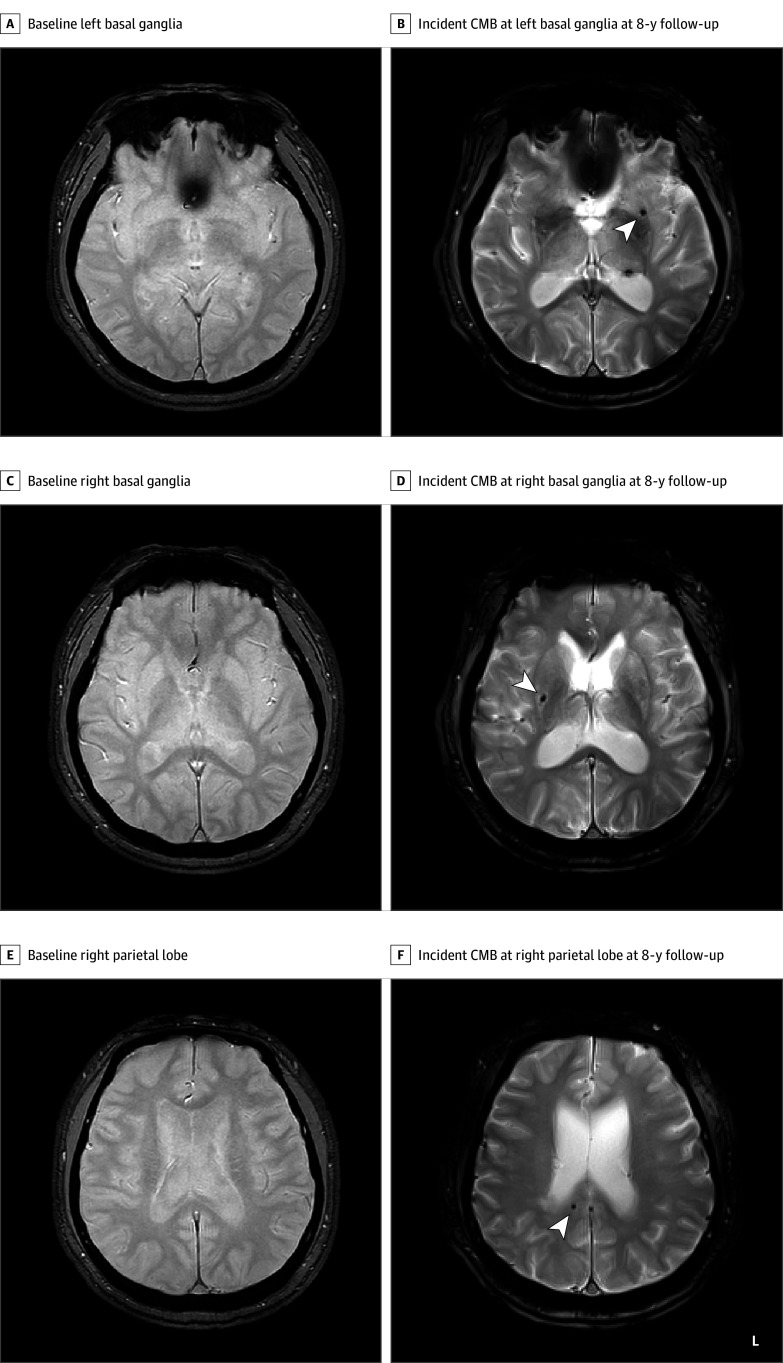
Cerebral Microbleed (CMB) Images at Baseline and Follow-Up Representation of CMBs of a 56-year old male participant in the Korean Genome and Epidemiology Study at baseline (A, C, E) and 8-year follow-up (B, D, F) by location on T2*-weighted gradient-recalled echo images. The participant had no CMBs at baseline (A, C, E) but had developed incident CMBs at left basal ganglia (B), right basal ganglia (D), and right parietal lobe (F) at follow-up. CMBs are indicated by the white arrows. L indicates left.

Details of the MRI procedure have been described elsewhere,^27,28^ and acquisition parameters specific to this study are given in the eMethods in Supplement 1. In short, scans were performed on a GE Signa 1.5T MR imaging scanner (GE Medical Systems) with an 8-channel head coil at baseline and first follow-up and with a Siemens Skyra 3T (Siemens Healthineers) scanner with a 64-channel head coil at second follow-up. Microbleeds were identified as well-defined, homogenous, hypointense lesions of less than 10 mm (commonly 2-5 mm) in diameter on T2*-weighted gradient-recalled echo images (Figure 2); these are most commonly located in the corticosubcortical junction and deep gray or white matter in the cerebral hemispheres, brainstem, and cerebellum. T2-weighted fluid-attenuated inversion recovery images were used to evaluate white matter changes (WMCs), appearing as areas of high signal intensities. Ill-defined hyperintensities of 5 mm or greater on fluid-attenuated inversion recovery images were identified as WMCs. The degree of WMC was scored using a 4-point age-related WMC (ARWMC) scale (0 = no lesion, 1 = focal lesion of ≤10 mm, 2 = beginning confluent lesions, and 3 = confluent lesions involving the entire region) in each right and left hemisphere.^29^ However, we used a dichotomized WMC variable (no lesion vs presence of focal or early confluence to diffusive lesions) given that most lesions were low-grade ARWMC in the study population.^28^ A trained radiologist who was blinded to the history and diagnosis of participant OSA status, scored CMBs and other radiological markers of small vessel disease (SVD) at all points. An intrarater reliability assessment was conducted across a 1-month interval with the data of a subsample of 56 participants, and results indicated a high repeatability (Cronbach α = 0.96).^30^

### Covariates

Questionnaire-based data were collected on demographic information, lifestyle, health status, and history of disease by trained examiners experienced in interviewing.^23^ Self-reported regular exercise (exercise >30-minutes ≥2 times per week) data were collected. Smoking and alcohol drinking status (never, former, or current) was determined by self-reported history. Body mass index (BMI) was calculated as weight in kilograms divided by height in meters squared. The change in BMI from baseline to follow-ups (change in BMI) was calculated (change in BMI = BMI at follow-up − BMI at baseline). Blood pressure was measured as the mean of the left and right arms using an appropriately sized cuff and a mercury sphygmomanometer (Baumanometer-Standby; W.A. Baum Co Inc) and mercury-free blood pressure monitor (BPBIO210T; Inbody) in a sitting position after the participant rested for at least 5 minutes. Mean arterial pressure was calculated using the following equation: (systolic blood pressure/3) + (diastolic blood pressure × 2/3). Plasma concentrations of glucose, total cholesterol, triglycerides, and high-density lipoprotein cholesterol were measured enzymatically using a 747 Chemistry Analyzer (Hitachi). Low-density lipoprotein cholesterol levels were estimated using the Friedewald formula. Type 2 diabetes was defined as a fasting glucose concentration of 126 mg/dL or greater or a glucose level 2 hours after the 75-g oral glucose tolerance test of 200 mg/dL or greater. Apolipoprotein E (APOE) genotypes were defined using rs429358 and rs7412 by Korea Biobank Array.^31^ *APOE-ε4* carriers were defined if they possessed at least 1 ε4 allele (ε2/ε4, ε3/ε4, or ε4/ε4).

### Statistical Analysis

All statistical analyses were performed using SAS statistical software version 9.4 (SAS Institute). Demographic, lifestyle, and clinical characteristics of study participants were expressed as mean (SD) or numbers and percentages stratified by OSA categories defined as no OSA (AHI level, 0-4.9 events/h), mild (AHI level, 5.0-14.9 events/h), and moderate to severe (AHI level, ≥15.0 events/h). We used 1-way analysis of variance for continuous variables and χ^2^ tests for categorical variables. Modified Poisson regression with robust error variance^32^ was used to estimate relative risk (RR), with 95% CIs, of incident CMBs (≥1 microbleed) in OSA groups, holding the non-OSA group as the reference category. Robust error variances were estimated using the repeated statement and subject identifier (subject ID) in the PROC GENMOD procedure in SAS. To account for within-participant correlation for participants, an unstructured correlation matrix was used. We examined univariate and multivariable models. In multivariable models, RRs were examined with adjustment for age, sex, and education. Other potentially confounding variables measured at baseline (BMI, physical activity level, smoking and drinking status, total and low-density lipoprotein cholesterol levels, hypertension status, diabetes status, and ARWMC status) were adjusted to determine if those variables interacted with the OSA-CMBs association. We ran the final model with additional adjustment for changes in AHI level (4-year and 8-year change in AHI), BMI (4-year and 8-year change in BMI), and blood pressure level (4-year and 8-year mean arterial pressure) over the follow-up. We performed sensitivity analyses after excluding participants who had a history of using continuous positive airway pressure (CPAP) during study follow-up. Finally, we performed a sensitivity analysis with additional adjustment for *APOE-ε4* status (carriers vs noncarriers) given that positive associations between *APOE-ε4* carrier status and CMBs have been reported previously.^33^ We considered 2-tailed *P* values < .05 to indicate statistical significance. Data were analyzed from March 2024 through January 2025.

## Results

### General Characteristics

Among 1441 study participants (mean [SD] age, 57.75 [5.53] at baseline; 759 female [52.67%]), 436 participants (30.25%) and 193 participants (13.39%) had mild and moderate to severe OSA, respectively, at baseline; 812 participants had no OSA. The moderate to severe OSA group had a higher proportion of men (136 men [70.47%] vs 226 men [51.83%]; *P* < .001) and participants who went to middle school or higher, although this difference was not significant (Table 1). Mean (SD) BMI was significantly higher in the moderate to severe (26.11 [3.18]) vs mild OSA (25.28 [2.72]) and non-OSA (23.94 [2.78]) groups. Regular exercise did not differ significantly among OSA groups. The moderate to severe OSA group had a higher proportion of current smokers and current drinkers. Among OSA groups, hypertension and diabetes were proportionately more frequent in the moderate to severe group. Mean (SD) serum total cholesterol levels differed significantly between moderate to severe and non-OSA groups (194.5 [41.1] mg/dL vs 202.8 [34.7] mg/dL) but not between moderate to severe and mild OSA groups. Among OSA groups, ARWMC level was proportionately higher in the moderate to severe group, although this difference was not significant (Table 1).

**Table 1.** Baseline General Characteristics of Study Participants

Characteristic	Participants, No. (%) (N = 1441)			*P* value^b^
	Non-OSA (n = 812)^a^	Mild OSA (n = 436)^a^	Moderate-Severe OSA (n = 193)^a^	
Age, mean (SD), y	56.80 (5.02)	58.80 (5.82)	59.36 (6.11)	<.001
Sex				
Women	492 (60.59)	210 (48.17)	57 (29.53)	<.001
Men	320 (39.41)	226 (51.83)	136 (70.47)	
≥Middle school education	550 (67.73)	284 (65.14)	138 (71.50)	.28
BMI, mean (SD)	23.94 (2.78)	25.28 (2.72)	26.11 (3.18)	<.001
Regular exercise	278 (34.24)	167 (38.30)	68 (35.23)	.35
Smoking status				
Never	549 (67.61)	264 (60.55)	95 (49.22)	<.001
Former	182 (22.41)	116 (26.61)	70 (36.27)	
Current	81 (9.98)	56 (12.84)	28 (14.51)	
Drinking status				
Never	419 (51.60)	201 (46.10)	65 (33.68)	<.001
Former	41 (5.05)	22 (5.05)	12 (6.22)	
Current	352 (43.35)	213 (48.85)	116 (60.10)	
Hypertension	258 (31.77)	195 (44.72)	114 (59.07)	<.001
Diabetes	172 (21.18)	135 (30.96)	79 (40.93)	<.001
Lipid levels, mean (SD), mg/dL				
Total cholesterol	202.8 (34.7)	198.3 (32.9)	194.5 (41.1)	.004
HDL cholesterol	50.4 (12.5)	47.8 (12.1)	44.3 (11.4)	<.001
LDL cholesterol	127.4 (31.9)	122.2 (29.3)	120.6 (35.3)	.002
Triglycerides	124.7 (62.0)	141.0 (70.5)	147.8 (71.6)	<.001
Presence of ARWMC	249 (30.67)	154 (35.32)	74 (38.34)	.06
*APOE-ε4* (n = 1233)				
Participants with data, No.	698	369	166	NA
Carrier status	126 (18.05)	76 (20.60)	32 (19.28)	.59
PSG recordings, mean (SD)				
TST, min	383.4 (76.0)	377.2 (76.3)	375.0 (77.7)	.22
Percentage of TST with Spo2 < 90%	0.14 (1.65)	0.82 (3.89)	3.67 (4.91)	<.001
Mean Spo2 percentage	96.16 (1.08)	95.36 (1.16)	94.66 (1.27)	<.001
Lowest Spo2 percentage	89.89 (5.24)	85.22 (4.78)	80.52 (6.39)	<.001
AHI, No. events/h	1.92 (1.42)	8.58 (2.62)	24.36 (10.14)	<.001
ODI, No. events/h	1.74 (1.36)	7.79 (2.87)	22.40 (9.94)	<.001

Abbreviations: AHI, apnea-hypopnea index; *APOE-ε4*, apolipoprotein E epsilon 4 allele; ARWMC, age-related white matter change; BMI, body mass index (calculated as weight in kilograms divided by height in meters squared); HDL, high-density lipoprotein; LDL, low-density lipoprotein; NA, not applicable; ODI, oxygen desaturation index; OSA, obstructive sleep apnea; Spo2, oxygen saturation measured by pulse oximetry; TST, total sleep time.

SI conversion factors: To convert total, HDL, and LDL cholesterol to millimoles per liter, multiply by 0.0259.

^a^ OSA categories are defined as non-OSA (AHI level, 0-4.9 events/h), mild OSA (AHI level, 5.0-14.9 events/h), and moderate to severe OSA (AHI level, ≥15.0 events/h).

^b^ *P* values are based on 1-way analysis of variance for continuous variables and χ^2^ tests for categorical variables.

### Incidence and Risk of CMBs

The cumulative incidence rate of CMBs in non-OSA, mild OSA, and moderate to severe OSA groups was 15 participants (1.85%), 7 participants (1.61%), and 9 participants (4.66%), respectively, at 4 years and 27 participants (3.33%), 14 participants (3.21%), and 14 participants (7.25%), respectively, at 8 years (Table 2). Most CMBs were single CMBs (eTable 1 in Supplement 1) and were mostly lobar by location in the brain (eTable 2 in Supplement 1). In crude Poisson models, the moderate to severe OSA group had an increased risk of developing CMBs compared with the non-OSA group at 4-year (RR, 2.52; 95% CI, 1.12-5.68; *P* = .02) and 8-year (RR, 2.18; 95% CI, 1.16-4.08; *P* = .01) follow-ups (Table 3). The associations remained in models adjusting for potential confounders. In multivariable models, after adjusting for age, sex, education level, BMI, physical activity level, smoking and drinking status, total and low-density lipoprotein cholesterol, hypertension, diabetes, and ARWMC level, we found that participants with moderate to severe OSA had increased risk of developing CMBs compared with the non-OSA group at 4-year (RR, 2.52; 95% CI, 1.07-5.92; *P* = .03) and 8-year (RR, 2.04; 95% CI, 1.04-3.99; *P* = .03) follow-ups (Table 3). However, in the final model with additional adjustment for change in AHI, change in BMI, and mean arterial pressure of the corresponding follow-up period, only 8-year risk remained significantly increased for the moderate to severe vs non-OSA group (RR, 2.14; 95% CI, 1.08-4.23; *P* = .02). No significant increased risks were observed in the mild OSA group at any points.

**Table 2.** Cumulative Incidence of CMBs

OSA category^a^	Participants, No.	CMB cumulative incidence rate, No. (%)	
		4 y	8 y
Non-OSA	812	15 (1.85)	27 (3.33)
Mild OSA	436	7 (1.61)	14 (3.21)
Moderate to severe OSA	193	9 (4.66)	14 (7.25)
All	1441	31 (2.15)	55 (3.82)

Abbreviations: CMB, cerebral microbleed; OSA, obstructive sleep apnea.

^a^ Non-OSA, mild OSA, and moderate to severe OSA are defined by apnea-hypopnea index levels of 0 to 4.9, 5.0 to 14.9, and 15.0 or more events/h, respectively.

**Table 3.** Association of OSA With CMB Risk (N = 1441)

OSA category^b^	Risk of CMBs							
	Model 1 (unadjusted)		Adjusted models^a^					
			Model 2		Model 3		Model 4	
	RR (95% CI)^c^	*P* value	RR (95% CI)^c^	*P* value	RR (95% CI)^c^	*P* value	RR (95% CI)^c^	*P* value
**4-y Follow-up**								
Non-OSA	1 [Reference]	NA	1 [Reference]	NA	1 [Reference]	NA	1 [Reference]	NA
Mild OSA	0.86 (0.35-2.11)	.75	0.76 (0.32-1.82)	.54	0.79 (0.31-1.99)	.62	0.77 (0.31-1.94)	.58
Moderate to severe OSA	2.52 (1.12-5.68)	.02	2.24 (1.01-4.98)	.04	2.52 (1.07-5.92)	.03	2.0 (0.78-5.14)	.14
**8-y Follow-up**								
Non-OSA	1 [Reference]	NA	1 [Reference]	NA	1 [Reference]	NA	1 [Reference]	NA
Mild OSA	0.96 (0.51-1.82)	.91	0.87 (0.46-1.63)	.67	0.89 (0.46-1.74)	.75	0.88 (0.44-1.74)	.72
Moderate-severe OSA	2.18 (1.16-4.08)	.01	1.89 (1.01-3.52)	.04	2.04 (1.04-3.99)	.03	2.14 (1.08-4.23)	.02

Abbreviations: OSA, obstructive sleep apnea; NA, not applicable; RR, relative risk.

^a^ Model 2 was adjusted for age, sex, and education level. Model 3 was adjusted for model 2 variables plus body mass index (BMI; calculated as weight in kilograms divided by height in meters squared), regular exercise, smoking and drinking status, total cholesterol, low-density lipoprotein cholesterol, hypertension, diabetes, and age-related white matter change. Model 4 was adjusted for model 3 variables plus change in AHI level (AHI at follow-up − AHI at baseline), change in BMI (BMI at follow-up − BMI at baseline), and mean arterial pressure of the corresponding follow-up.

^b^ OSA categories are defined as non-OSA (apnea-hypopnea index [AHI] level, 0-4.9 events/h), mild OSA (AHI level, 5.0-14.9 events/h), and moderate to severe OSA (AHI level ≥15.0 events/h).

^c^ RRs were estimated by Poisson regression with robust error variance.

### Sensitivity Analysis

We performed sensitivity analyses after excluding participants who had a history of using CPAP during study follow-up periods. A total of 21 participants reported using CPAP during the study period, although no compliance data were available. Results were essentially the same as in our main analysis (eTable 3 in Supplement 1). We also performed a sensitivity analysis by additionally adjusting for *APOE-ε4* genotype to check if there were any confounding effects due the genetic predisposition. The analysis was performed in a subsample of 1233 participants (Table 1) given that there were missing data for *APOE4* among 208 participants. We found that participants with moderate to severe OSA compared with the non-OSA group had an increased risk of developing CMBs at the end of the 8-year follow-up (RR, 2.91; 95% CI, 1.29-6.58; *P* = .01) with adjustment for *APOE4* genotype (eTable 4 in Supplement 1). The apparent higher magnitude of RRs in this analysis was likely due to the relatively smaller sample size than in the total sample that resulted in differential loss of participants with microbleeds from the non-OSA group vs OSA groups (eTable 5 in Supplement 1). Prevalence of the *APOE-ε4* allele in the sensitivity analysis population was low (234 participants with carrier status [18.98%], including 13 participants homozygous for ε4 (1.05%) (eTable 6 in Supplement 1).

## Discussion

To the best of our knowledge, this is the first prospective cohort study to report moderate to severe OSA as an independent risk factor associated with incident CMBs in a large general population of adults. We found that after adjusting for potential confounding factors, moderate to severe OSA was associated with increased risk of developing CMBs compared with the non-OSA group over an 8-year follow-up. However, no significant increased risk was observed over a 4-year follow-up. Mild OSA was not found to be associated with increased risk of CMBs at any follow-up period. Results were not altered by *APOE-ε4* genotype among study participants.

Previous studies have reported results of the association between OSA and CMBs. A meta-analysis^21^ found an odds ratio (OR) of 2.17 (95% CI, 0.61-7.73; *I*^2^ = 60.2%) for risk of CMBs in the moderate to severe OSA vs no OSA group, which was not a statistically significant difference. Two other meta-analyses^16,22^ investigating the association of OSA with cerebral SVD also did not find associations between OSA severity and CMBs. In a population-based study,^18^ an AHI level greater than 15 was associated with moderate to severe white matter hyperintensities but not with other neuroimaging signatures of SVD (namely, deep lacunar infarctions and deep CMBs), suggesting that OSA may be associated with diffuse subcortical brain damage of vascular origin but not focal lesions. In ethnically Korean populations, however, study findings on the association of CMBs with OSA remains mixed. One cross-sectional observational study^20^ demonstrated that a moderate to severe AHI level (≥15) was positively associated with the presence of CMBs in patients with OSA (OR, 4.51; 95% CI, 1.40-14.58; *P* = .01) after adjustment for potential confounding factors. However, in a study with 75 patients referred to a sleep center,^19^ the burden of CMBs was associated with AHI level, but no associations were observed between moderate to severe OSA and the existence of CMBs in the multivariable analysis (OR, 3.47; 95% CI, 0.89-15.18). However, both studies were performed using cross-sectional designs with relatively smaller sample sizes compared with our study.

The exact pathological mechanisms of the association of OSA with CMBs are not well known. One common mechanism could be hypertension, which is also associated with OSA, resultant of repeated episodes of hypoxia during sleep and the associated carotid body sensitization. Sleep apnea is also associated with blood pressure surges, which may be particularly injurious. However, even with adjustment for hypertension in our statistical models, the increased risk of CMBs associated with moderate to severe OSA remained. A few other mechanisms may also play a role in the occurrence of CMBs in association with OSA. Oxidative stress leads to production of free radicals, which may damage cell structure, including brain vasculature, potentially contributing to CMBs. Chronic hypoxia can also trigger inflammatory responses, leading to endothelial dysfunction and increased vascular permeability, making vessels more susceptible to bleeding.^34^ OSA has been found to be an independent risk factor associated with cardiac arrhythmias, especially atrial fibrillation (AF) and ventricular arrhythmias.^35^ OSA can predispose individuals to arrhythmia through several mechanisms, including intermittent hypoxia leading to increasing sympathetic nerve activity and increasing cardiac transmural pressure through negative intrathoracic force generated by inspiratory effort against a collapsed upper airway in OSA. One previous study found that patients with atrial fibrillation had a significantly higher prevalence of CMBs, and the presence of CMBs at baseline MRI was associated with the subsequent increase in CMBs in patients with AF.^36^ In our study, we excluded participants with baseline CMBs and a history of cardiovascular disease, including arrhythmias and still found an association between OSA and the risk of incident CMBs. This finding suggests that there may be other mechanisms involved in the association.

There is also a genetic predisposition to CMBs. *APOE-ε4* carrier status,^33^ a heritable component also associated with OSA, has been found to be associated with an increased number of microbleeds.^37^ In our study, the increased risk of CMBs in association with moderate to severe OSA remained significant even after adjustment for *APOE-ε4* genotype, indicating an independent association between OSA and occurrence of CMBs. However, the apparent increased magnitude of RRs, with wider CIs, in this sensitivity analysis was likely due to missing participants in the *APOE-ε4* analysis that resulted in exclusion of more individuals with microbleeds in the non-OSA group than OSA groups. Additionally, we observed very low prevalence of the *APOE-ε4* allele (18.98% carrier status with 1.05% homozygous) in our cohort compared with US and European cohorts, where *APOE-ε4* allele positivity was as high as 25% and 50%, respectively.^33,38^ This relatively low prevalence of *APOE-ε4* carrier status has been observed in the general population of Korea, as well as other Asian countries, such as Japan and China.^39^

### Limitations

This study has several limitations. First, the low prevalence of the *APOE-ε4* allele, with especially very few participants with homozygotes in our cohort, limited us to conduct further stratified analyses by genotype and OSA group in association with the occurrence of CMBs. Second, the number of incident cases of CMBs was not large enough to group them into meaningful categories (eg, lobar, deep, or infratentorial CMBs). Hence, our study could not disentangle whether OSA was associated with any particular spatial distribution of microbleeds. Similarly, almost all incident CMBs were single microbleeds, and therefore, we were unable to investigate the association of OSA severity with the degree of microbleed burdens. Third, our study used gradient-recalled echo sequences rather than the more sensitive susceptibility-weighted imaging sequences that are currently recommended for identification of CMBs. Therefore, there may be an underestimation of CMBs in our study. Fourth, we used a 1.5T MR imaging scanner at the baseline and first follow-up and a 3T scanner during the second follow-up. Previous studies suggested that detection rate and visibility of CMBs may benefit by up to 25% from the higher field strengths.^40,41^ In our study, however, the same radiologist read scans at all times. This may suggest that any increased detection of CMBs due to the 3T scanner at the second follow-up would likely happen randomly across all OSA groups and consequently not affect the strength of associations with incident microbleeds. Nonetheless, a potential scanner effect remains unexplored in this study. Fifth, we excluded participants from analysis due to deaths, dropouts, and missing data. Although our sample size is reasonably large, a potential selection bias cannot be completely ruled out. Sixth, a potential healthy cohort effect due to the long-term follow-up over 2 decades may limit the overall generalizability of our study findings.

## Conclusions

In this population-based prospective cohort study, moderate to severe OSA was independently associated with an increased risk of incident CMBs in the general adult population. Given that OSA is a modifiable risk factor, this finding suggests that moderate to severe OSA should be a potential target for early diagnosis and treatment to prevent incident CMBs and potentially prevent future strokes and dementia in aging populations.

## Notes

Supplement 1. **eTable 1.** Counts of cerebral microbleeds over follow-up periods **eTable 2.** Frequency distribution of cerebral microbleeds (CMBs) by location of microbleeds over the 8-year follow-up period of study participants (N = 1441) **eTable 3.** Relative risk (RR) of cerebral microbleeds (CMBs) by obstructive sleep apnea (OSA) categories over the 8-year follow-up period of the study participants (n = 1420) after excluding continuous positive airway pressure (CPAP) users **eTable 4.** Relative risk (RR) of cerebral microbleeds (CMBs) by obstructive sleep apnea (OSA) categories over the 8-year follow-up period of the study participants (n = 1233) with additional model adjustment for APOE4 genotype **eTable 5.** Cumulative incidence rate of cerebral microbleeds (CMBs) by severity of obstructive sleep apnea (OSA) in the subsample (n = 1233) **eTable 6.** Distribution of apolipoprotein E (APOE) genotypes across OSA groups
eMethods.

Supplement 2.
Data Sharing Statement
