# Error in Methods

**DOI:** 10.1001/jamanetworkopen.2025.54224

**Published:** 2025-12-22

In the Original Investigation titled “Obstructive Sleep Apnea and Cerebral Microbleeds in Middle-Aged and Older Adults,”^1^ published October 28, 2025, apolipoprotein E genotyping, done using the Korea Biobank Array, was incorrectly described as being done using restriction fragment length polymorphism analysis. The article has been corrected.^1^

## References

[1] Siddiquee AT, Hwang YH, Kim S, . Obstructive sleep apnea and cerebral microbleeds in middle-aged and older adults. JAMA Netw Open. 2025;8(10):e2539874. doi:10.1001/jamanetworkopen.2025.39874 41148136 PMC12569715

