# Supplementary material for: Obstructive Sleep Apnea and Cerebral Microbleeds in Middle-Aged and Older Adults
Source: JAMA Netw Open. 2025 Oct 28;8(10):e2539874. doi: 10.1001/jamanetworkopen.2025.39874 (PMC12569715; doi:10.1001/jamanetworkopen.2025.39874)
Supplement: Supplement 1. — eTable 1. Counts of cerebral microbleeds over follow-up periods eTable 2. Frequency distribution of cerebral microbleeds (CMBs) by location of microbleeds over the 8-year follow-up period of study participants (N = 1441) eTable 3. Relative risk (RR) of cerebral microbleeds (CMBs) by obstructive sleep apnea (OSA) categories over the 8-year follow-up period of the study participants (n = 1420) after excluding continuous positive airway pressure (CPAP) users eTable 4. Relative risk (RR) of cerebral microbleeds (CMBs) by obstructive sleep apnea (OSA) categories over the 8-year follow-up period of the study participants (n = 1233) with additional model adjustment for APOE4 genotype eTable 5. Cumulative incidence rate of cerebral microbleeds (CMBs) by severity of obstructive sleep apnea (OSA) in the subsample (n = 1233) eTable 6. Distribution of apolipoprotein E (APOE) genotypes across OSA groups eMethods. [file jamanetwopen-e2539874-s001.pdf]

Supplemental Online Content

Siddiquee AT, Hwang YH, Kim S, et al. Obstructive sleep apnea and cerebral microbleeds in middle-aged and older adults. *JAMA Netw Open*. 2025;8(10):e2539874. doi:10.1001/jamanetworkopen.2025.39874

eTable 1. Counts of cerebral microbleeds over follow-up periods

eTable 2. Frequency distribution of cerebral microbleeds (CMBs) by location of microbleeds over the 8-year follow-up period of study participants (N = 1441)

eTable 3. Relative risk (RR) of cerebral microbleeds (CMBs) by obstructive sleep apnea (OSA) categories over the 8-year follow-up period of the study participants (n = 1420) after excluding continuous positive airway pressure (CPAP) users

eTable 4. Relative risk (RR) of cerebral microbleeds (CMBs) by obstructive sleep apnea (OSA) categories over the 8-year follow-up period of the study participants (n = 1233) with additional model adjustment for APOE4 genotype

eTable 5. Cumulative incidence rate of cerebral microbleeds (CMBs) by severity of obstructive sleep apnea (OSA) in the subsample (n = 1233)

eTable 6. Distribution of apolipoprotein E (APOE) genotypes across OSA groups

This supplemental material has been provided by the authors to give readers additional information about their work.

© 2025 Siddiquee AT et al. *JAMA Network Open*.

eTable 1. Counts of cerebral microbleeds (CMBs) over follow-up periods

	4-year follow-up, n	***8-year follow-up, n***
Count of CMBs		
1 CMB	*29*	*49*
2 CMBs	*1*	*2*
>2 CMBs	*1*	*4*
Total (cumulative)	*31*	*55*

© 2025 Siddiquee AT et al. *JAMA Network Open.*

eTable 2. Frequency distribution of cerebral microbleeds (CMBs) by location of microbleeds over the 8-year follow-up period of the study participants (N=1441).

Location of CMBs	Brain areas	4-year follow-up, n (%)	8-year follow-up, n (%)
Lobar	Frontal lobe	8 (0.56)	19 (1.32)
	Temporal lobe	3 (0.21)	3 (0.21)
	Parietal lobe	8 (0.56)	15 (1.04)
	Occipital lobe	3 (0.21)	4 (0.28)
Deep	Basal ganglia	7 (0.49)	12 (0.83)
	Thalamus	1 (0.07)	3 (0.21)
Infra-tentorial	Brain stem	0	0
	Cerebellum	2 (0.14)	3 (0.21)

© 2025 Siddiquee AT et al. *JAMA Network Open*.

eTable 3: Relative risk (RR)[a] of cerebral microbleeds (CMBs) by obstructive sleep apnea (OSA) categories over the 8-year follow-up period of the study participants (N=1420) after excluding continuous positive airway pressure (CPAP) users.

	**Model 1** (Unadjusted model)		**Model 2** (adjusted for age, sex and education level)		**Model 3** (adjusted for model 2+ BMI, regular exercise, smoking, drinking, TC, LDL-C, hypertension, diabetes, and ARWMC)		**Model 4** (adjusted for model 3+ ΔAHI, ΔBMI and MAP of the corresponding follow-up)	
	RR (95% CI)	p-value	RR (95% CI)	p-value	RR (95% CI)	p-value	RR (95% CI)	p-value
**4-year follow-up**								
Non-OSA	1.0 [REF.]	NA	1.0 [REF.]	NA	1.0 [REF.]	NA	1.0 [REF.]	NA
Mild OSA	0.88 (0.36 - 2.14)	0.75	0.77 (0.32 - 1.84)	0.56	0.80 (0.32 - 2.0)	0.63	0.78 (0.31- 1.96)	0.60
Moderate-severe OSA	2.70 (1.20 - 6.07)	0.01	2.39 (1.08 - 5.32)	0.03	2.76 (1.16 - 6.55)	0.02	2.22 (0.84 - 5.81)	0.10
**8-year follow-up**								
Non-OSA	1.0 [REF.]	NA	1.0 [REF.]	NA	1.0 [REF.]	NA	1.0 [REF.]	NA
Mild OSA	0.98 (0.51 - 1.84)	0.95	0.88 (0.47 - 1.65)	0.70	0.91 (0.46 - 1.77)	0.78	0.90 (0.45 - 1.77)	0.76
Moderate-severe OSA	2.33 (1.25 - 4.36)	0.007	2.02 (1.08 - 3.76)	0.02	2.24 (1.13 - 4.43)	0.01	2.37 (1.18 - 4.75)	0.01

Abbreviations: OSA = Obstructive sleep apnea, AHI = Apnea–hypopnea index, ΔAHI = AHI at follow-up – AHI at baseline, BMI = Body mass index, ΔBMI = BMI at follow-up – BMI at baseline, MAP = Mean arterial pressure, TC = Total cholesterol, LDL-C = low-density lipoprotein cholesterol, ARWMC = age-related white matter change

OSA categories are defined as: Non-OSA (AHI 0–4.9 events/h), mild (AHI 5.0–14.9 events/h) and moderate-severe (AHI ≥15.0 events/h).

[a] Relative risks (RR) are estimated by Poisson regression with robust error variance.

© 2025 Siddiquee AT et al. *JAMA Network Open.*

eTable 4. Relative risk (RR)[a] of cerebral microbleeds (CMBs) by obstructive sleep apnea (OSA) categories over the 8-year follow-up period of the study participants (N=1233) with additional model adjustment for APOE4 genotype.

	**Model 1** (Unadjusted model)		**Model 2** (adjusted for age, sex and education level)		**Model 3** (adjusted for model 2+ BMI, regular exercise, smoking, drinking, TC, LDL-C, hypertension, diabetes, and ARWMC, APOE4 genotype)		**Model 4** (adjusted for model 3+ ΔAHI, ΔBMI and MAP of the corresponding follow-up)	
	RR (95% CI)	p-value	RR (95% CI)	p-value	RR (95% CI)	p-value	RR (95% CI)	p-value
**4-year follow-up**								
Non-OSA	1.0 [REF.]	NA	1.0 [REF.]	NA	1.0 [REF.]	NA	1.0 [REF.]	NA
Mild OSA	1.13 (0.41 - 3.09)	0.80	0.95 (0.35 - 2.59)	0.93	1.13 (0.39 - 3.24)	0.80	1.11 (0.39 - 3.17)	0.83
Moderate-severe OSA	3.36 (1.34 - 8.39)	0.009	2.84 (1.16 - 6.95)	0.02	3.72 (1.42 - 9.76)	0.007	2.91 (0.97 - 8.67)	0.05
**8-year follow-up**								
Non-OSA	1.0 [REF.]	NA	1.0 [REF.]	NA	1.0 [REF.]	NA	1.0 [REF.]	NA
Mild OSA	1.41 (0.67 - 2.96)	0.35	1.23 (0.59 - 2.58)	0.57	1.32 (0.61 - 2.88)	0.47	1.24 (0.56 - 2.74)	0.58
Moderate-severe OSA	3.41 (1.67 - 6.96)	<.001	2.84 (1.40 - 5.76)	0.003	3.17 (1.47 - 6.83)	0.003	2.91 (1.29 - 6.58)	0.01

Abbreviations: OSA = Obstructive sleep apnea, AHI = Apnea–hypopnea index, ΔAHI = AHI at follow-up – AHI at baseline, BMI = Body mass index, ΔBMI = BMI at follow-up – BMI at baseline, MAP = Mean arterial pressure, TC = Total cholesterol, LDL-C = low-density lipoprotein cholesterol, ARWMC = age-related white matter change

OSA categories are defined as: Non-OSA (AHI 0–4.9 events/h), mild (AHI 5.0–14.9 events/h) and moderate-severe (AHI ≥15.0 events/h).

[a] Relative risks (RR) are estimated by Poisson regression with robust error variance.

© 2025 Siddiquee AT et al. *JAMA Network Open.*

eTable 5. Cumulative incidence rate of cerebral microbleeds (CMBs) by severity of obstructive sleep apnea (OSA) in the sub-sample (N=1233).

OSA categories	Participants; n	4-year cumulative incidence rate; n (%)	8-year cumulative incidence rate; n (%)
Non-OSA	698	10 (1.43)	16 (2.29)
Mild OSA	369	6 (1.63)	12 (3.25)
Moderate-severe OSA	166	8 (4.82)	13 (7.83)
All	1233	24 (1.95)	41 (3.33)

Non-OSA, Mild OSA and Moderate-severe OSA are defined by apnoea–hypopnoea index 0–4.9, 5.0–14.9 and ⩾15.0 events/h, respectively.

© 2025 Siddiquee AT et al. *JAMA Network Open*.

eTable 6. Distribution of Apolipoprotein E (APOE) genotypes across the OSA groups.

APOE genotypes	Total (n=1233)	Non-OSA (n= 698)	Mild OSA (n=369)	Moderate-Severe OSA (n=166)
ε2/ε2	9 (0.73%)	6 (0.49%)	3 (0.24%)	0
ε2/ε3	148 (12%)	68 (5.52%)	52 (4.22%)	28 (2.27%)
ε2/ε4	15 (1.22%)	5 (0.41%)	8 (0.65%)	2 (0.16%)
ε3/ε3	842 (68.29%)	498 (40.39%)	238 (19.3%)	106 (8.60%)
ε3/ε4	206 (16.71%)	115 (9.33%)	64 (5.19%)	27 (2.19%)
ε4/ε4	13 (1.05%)	6 (0.49%)	4 (0.32%)	3 (0.24%)

Values are presented as n (%).

© 2025 Siddiquee AT et al. *JAMA Network Open*.

eMethods.

Polysomnography (PSG)

An unattended home-based PSG was performed with a portable device (Embletta X-100; Embla Systems, Broomfield, CO, USA) and data were collected in the morning after the overnight recordings. The following signals were documented: single-channel electroencephalogram (EEG) (C4-A1), electrooculogram (EOG), chin electromyogram (EMG), electrocardiography (EKG), airflow at the nose and mouth (using the pressure transducer airflow sensor), the chest and abdominal respiratory movement (respiratory impedance), oxygen saturation (pulse oximetry), and body position. PSG results were manually scored following most recent definitions for respiratory events according to the American Academy of Sleep Medicine guidelines. An apnea event was detected if both of the following criteria were met: (1) There was a drop in the peak signal excursion by ≥90% of the pre-event baseline (reference amplitude), (2) The duration of the ≥90% drop in sensor signal was ≥10 seconds. In addition, a hypopnea event was detected if all of the following criteria were met: (1) The peak signal excursions dropped by ≥30% of reference amplitude, (2) The duration of the ≥30% drop in signal excursions was ≥10 seconds, (3) There was ≥4% arterial oxygen desaturation from the reference amplitude or the event was associated with an arousal. The reference amplitude was calculated as the mean value of the peak amplitudes in the period of 100 seconds preceding the event. The apnea-hypopnea index (AHI) was calculated by averaging the total number of obstructive apnea and hypopnea events per hour of sleep.

MRI acquisition parameters:

The acquisition parameters of MRIs were as follows: GE 1.5T scanner with T2-weighted fluid-attenuated inversion recovery sequence (FLAIR; TR = 8802ms, TE = 129.06ms, TI = 2200ms, flip angle = 90°, matrix = 256 x 224, resolution = 0.86 x 0.98mm^2, slice thickness = 5mm) and T2*-weighted gradient-recalled echo sequence (GRE; TR = 450ms, TE = 15ms, flip angle = 26°, matrix = 256 x 224, resolution = 0.86 x 0.98mm^2, slice thickness = 5mm) and Siemens 3T scanner with FLAIR sequence (TR = 8910ms, TE = 90ms, TI = 2487ms, flip angle = 150°, matrix = 320 x 320, resolution = 0.7 x 0.7mm^2, slice thickness = 5mm) and GRE sequence (TR = 642ms, TE = 19.9ms, flip angle = 20°, matrix = 256 x 256, resolution = 0.9 x 0.9mm^2, slice thickness = 5 mm).

© 2025 Siddiquee AT et al. *JAMA Network Open*.
