# Supplementary material for: Obstructive Sleep Apnea and Cerebral Microbleeds in Middle-Aged and Older Adults
Source: JAMA Netw Open. 2025 Oct 28;8(10):e2539874. doi: 10.1001/jamanetworkopen.2025.39874 (PMC12569715; doi:10.1001/jamanetworkopen.2025.39874)
Supplement: Supplement 2. — Data Sharing Statement [file jamanetwopen-e2539874-s002.pdf]

Data Sharing Statement

Siddiquee. Obstructive Sleep Apnea and Cerebral Microbleeds in Middle-Aged and Older Adults. *JAMA Netw Open*. Published online October 28, 2025. doi:10.1001/jamanetworkopen.2025.39874

Data
Data available: Yes
Data types: Deidentified participant data, Data dictionary
How to access data: The data we used is not for public and can only be used (deidentified participant data) by those included in the IRB plan. Requests should be made to the Korean NIH if necessary (https://nih.go.kr/ko/main/contents.do?menuNo=300566). A contact can be made by the following email address: whalwls0227@korea.kr
When available: With publication

Supporting Documents
Document types: Statistical/analytic code, Informed consent form
How to access documents: The request can be sent to the corresponding author's email address as follows: chol-shin@korea.ac.kr
When available: With publication

Additional Information
Who can access the data: Data can be made available to researchers with an approved proposal.
Types of analyses: Data can be used for a specified purpose according to the approved proposal.
Mechanisms of data availability: Data can be made available to researchers with an approved proposal with investigator support.
Any additional restrictions: None
